# Arteriovenous fistulas in the craniocervical junction region: With vs. without spinal arterial feeders

**DOI:** 10.3389/fsurg.2022.1076549

**Published:** 2023-01-06

**Authors:** Zihao Song, Yongjie Ma, Yinqing Wang, Chuan He, Guilin Li, Peng Zhang, Tao Hong, Liyong Sun, Peng Hu, Ming Ye, Hongqi Zhang

**Affiliations:** ^1^ Department of Neurosurgery, Xuanwu Hospital, Capital Medical University, Beijing, China; ^2^ China International Neuroscience Institute (China-INI), Beijing, China

**Keywords:** arteriovenous fistula, arteriovenous shunt, craniocervical junction, spinal vascular malformation, subarachnoid hemorrhage

## Abstract

**Objective:**

Arteriovenous fistulas (AVFs) in the craniocervical junction (CCJ) region are a rare occurrence with special clinical manifestations. This study retrospectively reviewed patients with CCJ AVFs treated at our neurosurgical center, aiming to enhance the understanding of CCJ AVFs.

**Methods:**

A total of 113 patients with CCJ AVFs treated at our neurosurgical center between January 2013 and December 2020 were enrolled. They were grouped as patients with CCJ AVFs with spinal arterial feeders (*n* = 20) and patients with CCJ AVF without spinal arterial feeders (*n* = 93). Clinical presentation, angiographic characteristics, intraoperative findings, and treatment outcomes were analyzed.

**Results:**

The patients’ median age was 55 years (IQR 47.5–62 years). The proportion of males in the group without spinal arterial feeders was significantly higher (*p* = 0.001). Subarachnoid hemorrhage (SAH) was the most common clinical presentation, especially in the group with spinal arterial feeders (*p* < 0.001). There were significant differences in AVF type, fistula location, and direction of the venous drainage between the two groups (*p* < 0.001). Intervention embolization combined with microsurgery was more common in treating AVFs with spinal arterial feeders (*p* = 0.006). Spinal arterial feeders did not affect the outcome (*p* = 0.275).

**Conclusions:**

SAH was the most common presentation of CCJ AVFs in this study. Microsurgery and interventional embolization were optional treatment strategies. The angioarchitecture of CCJ AVFs was essential for selecting treatment strategies.

## Introduction

The craniocervical junction (CCJ), which includes the occipital bone and condyles, the atlas, and the axis, has unique and complex vascular and bone anatomy (1, 2). Arteriovenous fistulas (AVFs) in this region have complex anatomy and treatment. CCJ AVFs are rare vascular malformations found in less than 2% of patients with intracranial or spinal AVFs. In addition, this region of AVFs is quite different from other regions of spinal AVFs in terms of clinical characteristics, angioarchitecture, treatment modalities, and outcomes (3–6). In this study, we retrospectively reviewed 113 consecutive patients with CCJ AVFs treated at our neurosurgical center between 2013 and 2020 and divided these patients into two groups for subgroup analysis, i.e., those with and without spinal arterial feeders.

## Materials and methods

### Patients and follow-up

The study was approved by the medical ethics committee of our hospital. Informed consent was obtained from all patients. The data supporting the findings of this study were available from the corresponding author upon reasonable request.

This retrospective study included 113 patients with CCJ AVFs who were treated at our institution between January 2013 and December 2020. We collected patients’ clinical and radiological data, including baseline patient characteristics (age, sex, presentation), treatment strategies, outcomes [mRS (7) scores before and after treatment], and angiography files in the DICOM data format. SAHs were confirmed by computed tomography (CT) scanning of the head. For patients with Subarachnoid hemorrhage (SAH), we used the Hunt and Hess grading system (8). Venous hypertensive myelopathy (VHM) was defined as spinal cord edema and neurological dysfunction due to increased spinal venous pressure, which could present with motor and sensory dysfunction. AVFs were confirmed by DSA. Treatment strategies included microsurgery and embolization alone or a combination of the two. Intraoperative or postoperative angiography and intraoperative indocyanine green (ICG) angiography were used to confirm the obliteration of the fistula.

According to DSA and intraoperative sighting, each patient was diagnosed by two surgeons (neurosurgeons and neurointerventionalists with 20 years of experience in the field). Hiramatsu's (6) classification method was used with few modifications. In our definition, the location of the arteriovenous shunt was the standard of classification. Dural AVF (DAVF) is defined as the arteriovenous shunt located on the dural mater; radicular AVF (RAVF) is defined as the arteriovenous shunt located on the spinal nerve roots; epidural AVF (EDAVF) is defined as the arteriovenous shunt located outside the dura mater; perimedullary AVF (PAVF) is defined as the arteriovenous shunt located on the surface of the spinal cord.

Follow-up was performed by clinical examination or telephone interview. A total of 102 (90.3%) patients were followed up for at least 3 months, and 96 (85.0%) patients have been followed until now.

### Statistical analyses

SPSS 24.0 software (IBM Corp., Armonk, New York, USA) was used for statistical analysis. The *χ*^2^ test and the Fisher exact test were used to compare categorical variables of two groups, different clinical presentation groups, and different treatment groups, and the Wilcoxon signed-rank test was used to compare outcomes (mRS scores). Baseline patient characteristics, angiographic findings, and AVF types were assessed for association with SAH. Results are presented as relative risk with 95% CI. The potential risk factors with *p* < 0.10 on univariate analysis were included as confounders in the multivariate logistic regression model for multivariate analysis to determine whether or not they were risk factors for SAH. The variables of the final model were selected by the forward conditional method. Results are presented as odds ratios with 95% CI. Statistical analyses were two-sided, with a *p* < 0.05 considered statistically significant.

## Results

### Clinical characteristics

The clinical characteristics data of two groups of patients are listed in Table 1. There were 93 (82.3%) male CCJ AVF patients, and the proportion of males in the group without spinal arterial feeders was higher (*p* = 0.001). The median age at presentation of overall patients was 55 years [interquartile range (IQR), 47.5–62 years]. SAH was the most common clinical presentation, especially in the group with spinal arterial feeders (*p* < 0.001). For patients with SAH, 63 (96.9%) patients presented with mild neurological dysfunction (Hunt and Hess grade 1–3). There was no significant difference in mRS scores between the two groups before the operation (*p* = 0.313).

**Table 1:** Clinical characteristics according to spinal arterial feeders.

	Spinal arterial feeders		Total	*p-*value
	With	Without		
No. of patients	20	93	113	
No. of lesions	24	98	122	0.083
Male sex	11 (55.0)	82 (88.2)	93 (82.3)	**0** **.** **001**
Median age (IQR)	51 (45-58)	56 (48-62)	55 (47.5-62)	0.058
Presentation			** **	**<0.001**
SAH	19 (95.0)	46 (49.5)	65 (57.5)	
VHM	1 (5.0)	47 (50.5)	48 (42.5)	
Hunt and Hess grade				0.051
1	3 (15.8)	13 (28.3)	16 (24.6)	
2	12 (63.2)	32 (69.6)	44 (67.7)	
3	3 (15.8)	0 (0)	3 (4.6)	
4	1 (4.3)	1 (2.2)	2 (3.1)	
5	0 (0.0)	0 (0)	0 (0)	
Pretreatment mRS				0.313
0	0 (0.0)	0 (0)	0 (0)	
1	2 (10.0)	26 (28.0)	28 (24.8)	
2	11 (55.0)	38 (40.9)	49 (35.4)	
3	2 (10.0)	0 (9.7)	2 (1.8)	
4	1 (5.0)	12 (12.9)	13 (11.5)	
5	5 (20.0)	17 (18.3)	21 (18.6)	

Bold text indicates statistical significance.

### Angioarchitecture

As shown in Table 2, there were 122 fistulas in 113 patients. A total of 104 (92.0%) patients had a single fistula, and 9 (8.0%) patients had dual fistulas. Among the 24 fistulas with spinal arterial feeders, 20 (83.3%) fistulas were fed by the anterior spinal artery (ASA) and 9 (37.5%) fistulas were fed by the lateral spinal artery (LSA). Overall, the most common CCJ AVF type was DAVF, with 87 fistulas (71.3%). The proportion of CCJ AVF type was significantly different among the two groups (*p* < 0.001). For CCJ AVFs with spinal arterial feeders, the most common CCJ AVF type was RAVF, with 15 (62.5%) fistulas. In contrast, there was no RAVF or PAVF in CCJ AVFs without spinal arterial feeders.

**Table 2:** Angioarchitecture according to spinal arterial feeders.

	Spinal arterial feeders		Total	*p*-Value
	With	Without		
No. of patients	20	93	113	
No. of lesions	24	98	122	
AVF type				**<0** **.** **001**
DAVF	1 (4.2)	86 (87.8)	87 (71.3)	**<0**.**001**
RAVF	15 (62.5)	0 (0.0)	15 (12.3)	**<0**.**001**
EDAVF	2 (8.3)	12 (12.2)	14 (11.5)	0.856
PAVF	6 (25.0)	0 (0.0)	6 (4.9)	**<0**.**001**
Side				0.570
Left	12 (50.0)	54 (55.1)	66 (54.1)	0.653
Right	12 (50.0)	41 (41.8)	53 (43.4)	0.470
Both	0 (0.0)	3 (3.1)	3 (2.5)	1.000
Fistula location				**<0**.**001**
FM	0 (0.0)	6 (6.1)	6 (4.9)	0.597
C-1	12 (50.0)	79 (80.6)	91 (74.6)	**0**.**002**
C-2	12 (50.0)	13 (13.3)	25 (20.5)	**<0**.**001**
Direction of the venous drainage				**<0.001**
Intradural	18 (75.0)	91 (92.9)	109 (89.3)	**0**.**030**
Ascending intradural	17 (70.8)	47 (48)	64 (52.5)	**0**.**044**
Descending intradural	2 (8.3)	58 (59.2)	60 (49.2)	**<0**.**001**
Epidural	7 (29.2)	8 (8.2)	15 (12.3)	**0**.**014**
Aneurysmal structure	14 (58.3)	18 (18.4)	32 (26.2)	**<0.001**
Varix	19 (79.2)	62 (63.3)	81 (66.4)	0.139

FM, foramen magnum; C, cervical.

Bold text indicates statistical significance.

On the side of the lesion, CCJ AVFs with spinal arterial feeders were equally distributed on the left and right, while CCJ AVFs without spinal arterial feeders were more distributed on the left, and three (3.1%) fistulas were fed by both vertebral arteries. CCJ AVFs with spinal arterial feeders showed no tendency in fistula location, while most of the CCJ AVFs without spinal arterial feeders were located at the C-1 level, with 79 (80.6%) fistulas, and 6 (6.1%) fistulas were located at the level of the foramen magnum. There were differences in the location of the fistula between the two groups (*p* < 0.001).

There were 109 (89.3%) fistulas with intradural drainage and 15 (12.3%) fistulas with epidural drainage. The draining veins of CCJ AVFs without spinal arterial feeders were more inclined to drain to intradural, especially descending intradural (*p* = 0.030 and *p* < 0.001, respectively). Aneurysmal structures occurred at 32 (26.2%) fistulas, and CCJ AVFs with spinal arterial feeders seemed more likely to form aneurysmal structures (*p* < 0.001). Varices of draining veins occurred at 81 (66.4%) fistulas.

### Treatment and outcomes

**Figure 1:**
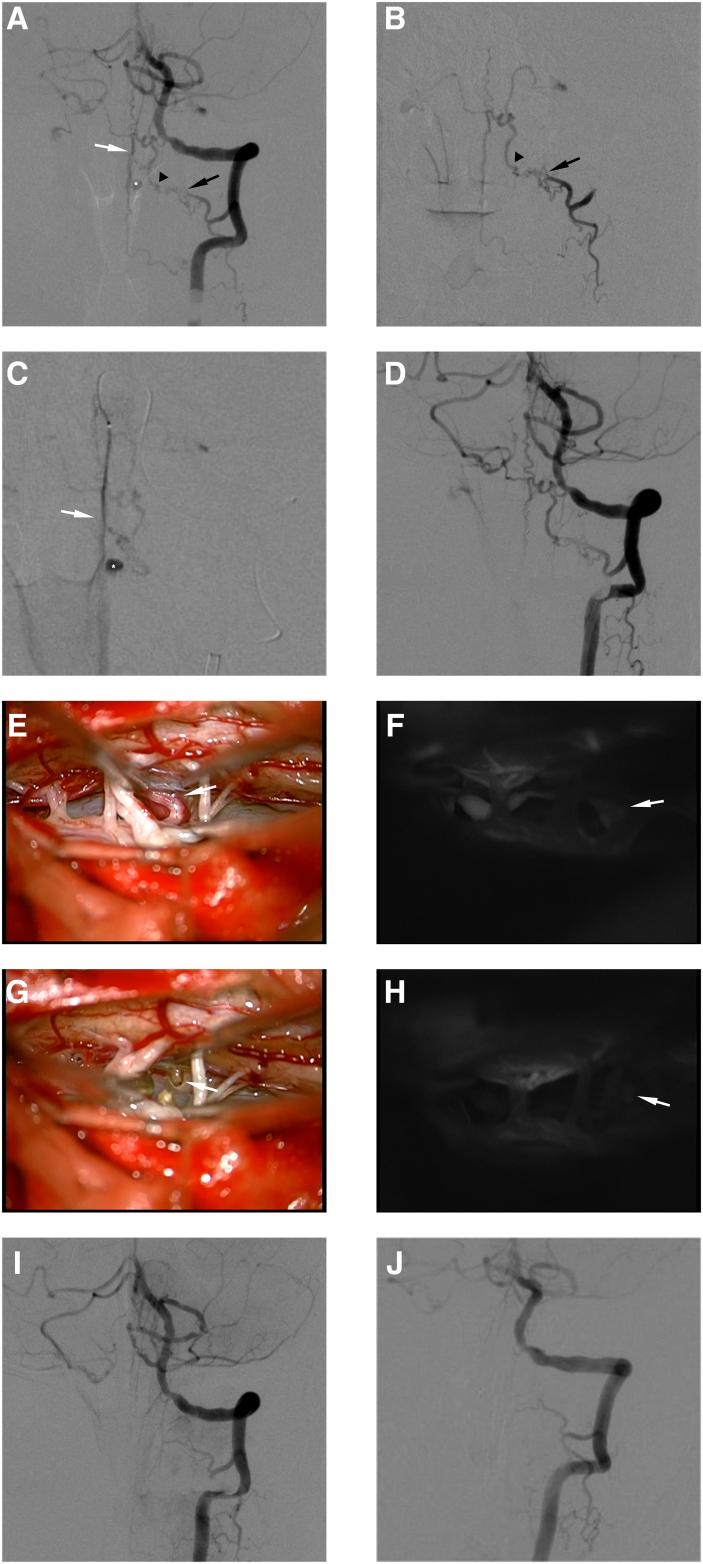
This female RAVF patient presented with SAH and was treated with interventional embolization and microsurgery. Preoperative angiography of left VA (**A**). Superselective angiography of RA (**B**). Superselective angiography of ASA (**C**). The AVF was fed by left C-2 RA (black arrows in **A,B**) and ASA (white arrows in **A,C**), and the feeder drained into the intradural vein (black arrowhead in **A**). The draining vein formed an aneurysmal structure (white asterisks in **A,C**). Postembolization angiography of left VA (**D**). We blocked blood supply to the aneurysmal structure by placing a coil in ASA. The intraoperative image showed that the draining vein was closely related to the nerve root (**E**). Indocyanine green angiography showed that the draining vein drains into the cephalic side (**F**). The same position of the draining vein was marked (white arrows in **E,F**). The intraoperative image showed that the draining vein and nerve root were disconnected (**G**). Indocyanine green angiography showed the disconnection of the AVF (**H**). The same position of the disconnected draining vein was marked (white arrows in **G,H**). Postoperative angiography 5 days after microsurgery of left VA (**I**). Postoperative angiography 3 months after microsurgery of left VA (**J**).

**Figure 2:**
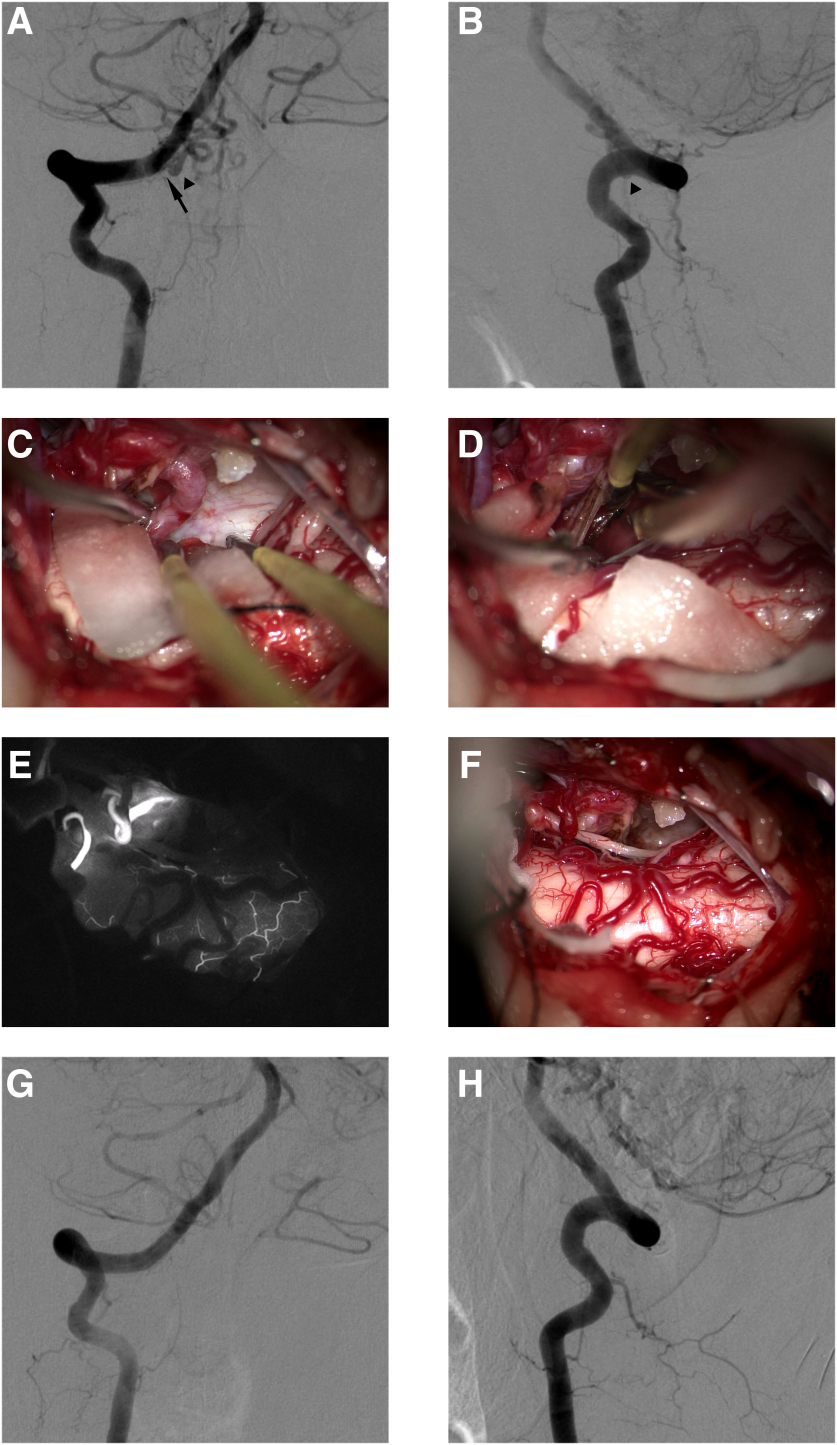
This female DAVF patient presented with VHM and was treated with microsurgery. Preoperative anteroposterior angiography of right VA (**A**). Preoperative lateral angiography of right RA (**B**). The AVF was fed by the right C-1 dural branch of VA (black arrow in **A**), and the feeder drained into the intradural vein (black arrowheads in **A,B**). The intraoperative image showed that the draining vein drains into the cephalic side (**C**). The intraoperative image showed that the draining vein was disconnected under the occlusion of the aneurysm clip (**D**). Intraoperative image (**E**) and indocyanine green angiography (**F**) in the same perspective showed the disconnection of the AVF. Postoperative anteroposterior angiography 4 days after microsurgery of right VA (**G**). Postoperative lateral angiography 4 days after microsurgery of right VA (**H**).

Treatment and outcome information is listed in Table 3. Microsurgery was the most common treatment strategy, as 98 (80.4%) fistulas were cured only by microsurgery. Interventional embolization and microsurgery were used to treat 17 (13.9%) fistulas (Figure 1). There were also differences in the choice of treatment strategies between the two groups (*p* = 0.009). Microsurgery was more common in the treatment of CCJ AVFs without spinal arterial feeders (*p* = 0.030) (Figure 2), and interventional embolization combined with microsurgery was more common in the treatment of CCJ AVFs with spinal arterial feeders (*p* = 0.006). All patients were evaluated by angiography or intraoperative indocyanine green angiography for AVFs disconnected.

**Table 3:** Treatment and outcome according to spinal arterial feeders.

	Spinal arterial feeders		Total	*p*-Value
	With	Without		
No. of patients	20	93	113	
No. of lesions	24	98	122	
Treatment			** **	**0.009**
Surgery	15 (62.5)	83 (84.7)	98 (80.4)	**0**.**030**
Embolization	1 (4.2)	6 (6.1)	7 (5.7)	1.000
Embolization + surgery	8 (33.3)	9 (9.2)	17 (13.9)	**0**.**006**
Complications	6 (25.0)	16 (16.3)	22 (18.0)	0.487
Intracranial infection	6 (25.0)	6 (6.1)	12 (9.8)	**0**.**016**
Pulmonary infection	0 (0.0)	3 (3.1)	3 (2.5)	1.000
CSF leak	0 (0.0)	3 (3.1)	3 (2.5)	1.000
Spinal infarction	0 (0.0)	1 (1.0)	1 (0.8)	1.000
Cerebral infarction	0 (0.0)	1 (1.0)	1 (0.8)	1.000
VA occlusion	0 (0.0)	1 (1.0)	1 (0.8)	1.000
Cranial nerve paralysis	0 (0.0)	1 (1.0)	1 (0.8)	1.000
Post-treatment mRS^a^				0.275
0	6 (30.0)	29 (31.2)	35 (31.0)	
1	12 (60.0)	35 (37.6)	47 (41.6)	
2	1 (5.0)	9 (9.7)	10 (8.8)	
3	0 (0.0)	0 (0.0)	0 (0.0)	
4	1 (5.0)	12 (12.9)	13 (11.5)	
5	0 (0.0)	4 (4.3)	4 (3.5)	
6	0 (0.0)	4 (4.3)	4 (3.5)	

CSF, cerebrospinal fluid.

Bold text indicates statistical significance.

^a^ Post-treatment mRS was evaluated according to the last follow-up.

The incidence of postoperative complications was 18.0%, and there was no significant difference between the two groups. The most common complications were infection, and all infected patients recovered well. Cerebrospinal fluid (CSF) leak occurred in three (2.5%) patients. Spinal infarction occurred in one (0.8%) patient; during microsurgery, AVFs ruptured and bled again. After strict hemostasis, spinal cord infarction occurred, and the patient's lower limb muscle strength decreased. Cerebral infarction occurred in one (0.8%) patient; this patient developed cerebral vasospasm and cerebral infarction on the 8th day after microsurgery, later underwent decompressive craniectomy, and died after 1 month. In one (0.8%) patient, the VA was occluded on the side of the lesion due to treatment requirements, and the patient suffered from intermittent dizziness after occlusion. Cranial nerve paralysis occurred in one (0.8%) patient who presented with numbness of the tongue root.

The median mRS scores of pretreatment and post-treatment were 2 and 1. The mRS scores improved after treatment (*p* < 0.001). After treatment, 92 (81.4%) patients had good outcomes (mRS scores 0–2). Spinal arterial feeders did not affect the outcome (*p* = 0.275).

### Risk factors associated with SAH

As shown in Table 4, we used univariate and multivariate analyses to identify risk factors associated with SAH. In univariate analysis, risk factors included age ≤55, RAVF, EDAVF, with spinal feeders, ascending intradural drainage, epidural drainage, and aneurysmal structure. After using the forward conditional method in in multivariate analysis, DAVF [odds ratio (OR) 0.104, 95%CI: 0.023–0.475, *p* = 0.003], ascending intradural drainage (OR: 4.684, 95%CI: 1.445–15.180, *p* = 0.010), and descending intradural drainage (OR: 0.296, 95%CI: 0.088–0.988, *p* = 0.048) were significantly associated with SAH.

**Table 4:** Risk factors associated with SAH.

Variable	Univariate			Multivariate		
	RR	95%CI	*p-*Value	OR	95%CI	*p-*Value
Sex: male	0.738	0.556–0.979	0.078			
Age ≤55	1.406	1.039–1.903	**0** **.** **022**			** **
Left side	0.856	0.645–1.136	0.286			
Right side	1.058	0.795–1.407	0.701			
C-1	0.860	0.638–1.159	0.860			
C-2	1.340	1.012–1.775	0.078			
FM	0.537	0.172–1.680	0.329			
DAVF	0.528	0.415–0.671	**<0**.**001**	0.104	0.023–0.475	**0**.**003**
RAVF	1.664	1.342–2.065	**0**.**006**			
EDAVF	1.493	1.141–1.953	**0**.**041**			
PAVF	1.706	1.464–1.988	0.111			
With spinal feeders	1.842	1.496–2.266	**<0**.**001**			
Intradrain drainage	0.616	0.491–0.773	**0**.**013**			
Ascending intradural drainage	2.010	1.429–2.825	**<0**.**001**	4.684	1.445–15.180	**0**.**010**
Descending intradural drainage	0.383	0.264–0.554	**<0**.**001**	0.296	0.088–0.988	**0**.**048**
Epidural drainage	1.664	1.342–2.065	**0**.**006**			
Aneurysmal structure	1.616	1.261–2.070	**0**.**001**			
Varix	0.785	0.595–1.036	0.105			

RR, relative risk; OR, odds ratio; FM, foramen magnum; C, cervical; RAVF, radicular arteriovenous fistula; PAVF, perimedullary arteriovenous fistula; EDAVF, epidural arteriovenous fistula; DAVF, dural arteriovenous fistula.

Bold text indicates statistical significance.

## Discussion

In the previous study, we carefully explored the classification and treatment strategies of CCJ AVFs with spinal arterial feeders using our single-center data (9). In the present study, we divided the CCJ AVFs into two groups according to whether they were with or without spinal arterial feeders to explore the differences between the two groups in clinical presentation and treatment strategies. In addition, we tried to find the risk factors for SAH, hoping to help our colleagues understand and treat CCJ AVFs through the experience of our center.

### Clinical characteristics and angioarchitecture

In the previous systematic review, CCJ AVFs often occurred in middle age, and the male-to-female ratio was about 3:1 (5, 10). In the present study, the proportion of male patients was higher than previously reported in the group without spinal arterial feeders and total patients, while in the group with spinal arterial feeders, the proportion of male and female patients was almost equal.

Previous studies have shown that VHM and SAH are the most common clinical presentation of CCJ AVF patients (6, 11, 12). The proportion of the two clinical presentations in the group without spinal arterial feeders was equal. In the group with spinal arterial feeders, the incidence of SAH was significantly higher than that of VHM, which prompted us to explore the risk factors for SAH. In early studies, the direction of venous drainage was often considered a factor of different clinical presentations (5, 10, 13, 14). With a better understanding of the angioarchitecture of CCJ AVFs, aneurysmal structure, arterial feeder of ASA, and even different AVF types (e.g., RAVF) were considered as high-risk factors for SAH (6, 15). Our univariate analysis further confirmed that these were risk factors for SAH. In multivariate analysis, ascending intradural drainage was the most relevant risk factor for SAH. Compared to normal venous flow, the ascending intradural drainage could be regarded as high flow reflux, while descending intradural drainage only increased flow, which should explain the high probability of SAH in CCJ AVFs with ascending intradural drainage. However, with spinal feeders was not considered as a risk factor in multivariate analysis. More studies were needed to explore whether there was correlation between spinal feeders and SAH.

### Treatment and outcomes

According to the experience of previous studies, microsurgery and interventional embolization were optional treatment strategies for CCJ AVFs. Nevertheless, like AVFs in other parts of the spinal cord, microsurgery was favored for CCJ AVFs, and it usually resulted in better outcomes (5, 11, 16–19). According to the treatment experience of our center, the angioarchitecture of CCJ AVFs was the key to our choice of treatment strategies.

Simple microsurgical treatment was more appropriate for lesions without spinal feeders and those mainly located in the dorsal or lateral position. According to the dorsal or lateral position of the lesion, the posterior median approach and the far lateral approach were selected. If the lesion only had a single draining vein, we preferred to electrocoagulate and disconnect the draining vein. In treating AVF with multiple arterial feeders and drainage varices, test occlusion and intraoperative ICG fluorescence angiography were necessary to determine the location of the shunt and abnormal blood vessels (11, 20, 21). Compared with interventional embolization, which might lead to false embolism and cause brainstem infarction, microsurgery was often safer and more reliable. For microsurgery in the CCJ region, our center also routinely used intraoperative neurophysiological monitoring to help preserve neurophysiological function. After drainage veins were disconnected, it was necessary to perform ICG fluorescence angiography or intraoperative DSA to ensure that the lesion was completely removed. If intraoperative angiography could not be performed, it was necessary to perform postoperative DSA in time.

For lesions with spinal feeders or those mainly located in the ventral position, the architecture was more complex and often formed aneurysmal structures. In Takai's study, for lesions with an aneurysmal structure or varix, if only the AVF was treated, the aneurysmal structure or varix might spontaneously shrink (22), which might provide a new way for the treatment of such complex CCJ AVF. However, in the absence of additional evidence, we preferred disconnecting the drainage veins and handling the aneurysmal structure. During microsurgery, it is almost impossible to clip the ventral aneurysm or disconnect the ventral drainage vein. Therefore, for these CCJ AVFs, we often performed interventional embolization of abnormal ventral vessels first, as it was relatively safe to embolize near the fistula from the ASA. However, if we chose to embolize from radicular artery, glue might enter the ASA or LSA through radiculopial artery or radiculomedullary artery and cause ischemic events (22–24). Nevertheless, because ASA was very thin and at an acute angle with the vertebral artery, it was often difficult to put the microcatheter in place. For this reason, one patient only received microwire electrocoagulation during interventional therapy. Due to the complex vascular anatomy in this region, adhesive glue was safer and more reliable than nonadhesive glue, and we preferred n-BCA or Glubran 2 (GEM Srl, Viareggio, Italy) to Onyx (Medtronic, CA, USA). In a meta-analysis of outcomes following surgical vs. endovascular treatment of spinal DAVF, Onyx was associated with significantly higher odds of initial failure or late recurrence than n-BCA (OR: 3.87, 95%CI: 1.73–8.68, *I*^2^: 0%, *p* < 0.001) (25). Similarly, n-BCA was also most commonly used in the research of Yoo (26); yet, ischemic events occurred in six (46.2%) patients. Therefore, the prevention of ischemic events during embolization was an important step.

In the present study, the probability of postoperative complications was 18.0% (22/122), and the most common complications were infections. The incidence of intracranial infection was higher in the group with spinal arterial feeders (*p* = 0.016), which might be related to the long microsurgery time due to the complex angioarchitecture. Notably, in the group with spinal arterial feeders, no patients had infarction-related complications, which might be related to more patients in this group undergoing both interventional embolization and microsurgery. In a recent multicenter study, the complications’ overall incidence was 26% (25/97). Ischemic complications were the most common complications, and the associated risk factors were interventional embolization (OR: 4.3, 95%CI: 1.1–16, *p* = 0.030) and spinal arterial feeders (OR: 3.8, 95%CI: 1.03–14, *p* = 0.045) (19, 22). In treating CCJ AVFs with complex angioarchitecture, it was necessary to carefully identify the blood vessels to avoid ischemic events, especially during interventional embolization.

In previous studies, younger age (*p* = 0.043) and hemorrhagic presentation (*p* = 0.002) were significant predictors of better outcomes (5). Also, the type of CCJ AVF was a factor affecting the outcome. mRS scores could improve significantly (*p* = 0.0389) in patients with DAVF (6). A recent study showed that poor outcomes were associated with a pretreatment mRS score ≥3 (OR: 13, 95%CI: 2.7–62, *p* = 0.001) and complications (OR: 5.8, 95%CI: 1.3–26, *p* = 0.020), while spinal arterial feeders was not a significant factor (*p* = 0.64) (19). In the present study, spinal arterial feeders had a greater influence on the clinical presentation and the choice of treatment strategies and had no significant impact on the outcomes.

### Study limitations

The present study also has several limitations. First, this was a retrospective single-center study. The experience of our center may not necessarily apply to other centers. Second, this study mainly used mRS scores for outcome ranks, which may not be sufficient to reflect subtle changes. Third, incorrect mRS scores also may occur, especially during telephone interviews. Thus, prospective multicenter studies should be conducted to further confirm these findings and guide clinical treatment.

## Conclusions

This study divided CCJ AVFs into two groups according to whether they were with or without spinal arterial feeders. SAH was the most common presentation of CCJ AVFs. Microsurgery and interventional embolization were optional treatment strategies. The angioarchitecture of CCJ AVF was essential to our choice of treatment strategies. As microsurgery was often safer and more reliable, it could be the first treatment choice.

## Data Availability

The original contributions presented in the study are included in the article/Supplementary Material; further inquiries can be directed to the corresponding authors.
